# The Association of *OTX1* rs17850223 Polymorphisms in Han Chinese Patients with Idiopathic Epilepsy

**DOI:** 10.1155/2020/4375293

**Published:** 2020-03-10

**Authors:** Jin Lv, Chunsheng Qu, Zhenqiang Huang, Yingbiao Zhu, Wei Wang, Likang Lan

**Affiliations:** ^1^Department of Neurology of Lishui People's Hospital, The Sixth Affiliated Hospital of Wenzhou Medical University, Lishui, Zhejiang 323000, China; ^2^Clinical Laboratory of Lishui People's Hospital, The Sixth Affiliated Hospital of Wenzhou Medical University, Lishui, Zhejiang 323000, China

## Abstract

This study is aimed at investigating the association between orthodenticle homeobox 1 (*OTX1*) gene polymorphisms and idiopathic epilepsy in a cohort of Han Chinese patients. We carried out a case-control study on 147 patients with idiopathic epilepsy and 150 healthy controls. Genomic DNA was isolated from 1 ml of ethylene diamine tetraacetic acid (EDTA)-treated blood. The *OTX1* coding sequence was divided into three parts and amplified using PCR, and the products were genotyped using the Sanger sequencing method. All *OTX1* coding sequences were conserved except for rs17850223 located on the fifth exon. The frequency of the CC, CG, and GG genotypes showed no statistical differences between the idiopathic epileptic patients and the controls. The rs17850223 G allele distribution was also similar between the idiopathic epileptic patients and the controls. Interestingly, the frequency of the GG genotype was significantly higher in the patients with generalized seizures compared with that of the controls (12.2% vs. 2%, *p* = 0.012), and a greater distribution of the rs17850223 G allele was also seen in the patients with generalized seizures compared with controls (18.3% vs. 10%, *p* = 0.049). rs17850223 might play a critical role in Chinese idiopathic epileptic patients with generalized seizure activity.

## 1. Introduction

Epilepsy is a chronic episodic noncommunicable disorder of the brain due to abnormal excessive or synchronous neuronal activity. This disorder affects about 50 million people worldwide, and idiopathic epilepsy represents the majority of all epileptic seizure disorders [1–3]. There is increasing evidence that demonstrates genetic factors play a crucial role in the development of idiopathic epilepsy; these findings are mostly based on epidemiological studies, including those of familial aggregation, monozygotic twins, and families with a history of epilepsy [4–6].

Orthodenticle homeobox 1 (*OTX1*) is one of the homeobox genes that belongs to the *OTX* family (*OTX1*, *OTX2*, *OTX3*, and *CRX*) [7, 8]. The *OTX1* gene encodes transcription factors that have high affinity for TAATCC/T elements on target genes [9] involved in brain regionalization, corticogenesis, and organ development sense during embryogenesis [10]. *OTX1* expression gradually declines but remains at detectable levels in the deep cortical layers of mature rats [11]. The *OTX1* mutant mice showed a circle-running behavior, which exhibited spontaneous epileptic seizures exemplified by upper extremity clonus, rearing, falling, and convulsions starting at the fourth postnatal week [12]. In these mice, neuronal populations in the cortex displayed abnormal electrical activities during convulsive seizures [13].

The main objective of this study was to assess the polymorphisms of the *OTX1* coding sequences in a cohort of patients from China based on the hypothesis that these polymorphisms are potentially predictive of epilepsy.

## 2. Patients and Methods

### 2.1. Patients

A total of 147 patients with idiopathic epilepsy and 121 healthy individuals were consecutively recruited in our hospital from July 2015 to July 2018. All patients fulfilled the requirements of the International League Against Epilepsy classification for idiopathic epilepsy [14]. Patients with other comorbid conditions that might have been associated with brain structure alterations, such as intracranial tumors, cerebrovascular disease, metabolic encephalopathy, dementia, language disabilities, and activity disorders, were excluded. None of the control participants had a history of a central nervous system disorder or any other medical disorders.

### 2.2. DNA Extraction and Genotyping

**Figure 1:**
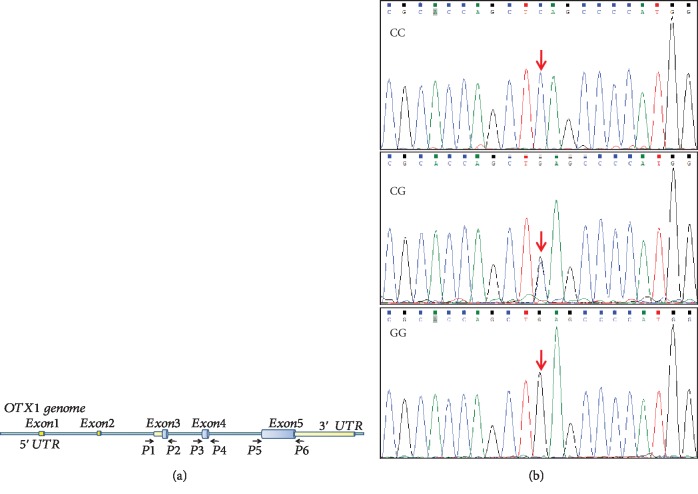
(a) The coding sequence of *OTX1*. (b) Gene sequencing diagram of rs17850223 polymorphism; the red arrow showed the mutation site.

We purified genomic DNA from EDTA-anticoagulated blood samples using the QIAamp DNA Mini Kit (Qiagen, lot no. 51304). After DNA extraction, the concentration and purity of DNA were detected using the NenoDrop2000 (Thermo). DNA with a 260/280 ratio value of approximately 1.8 and a concentration of greater than 50 ng/ml were accepted. The primer sequences were designed using Primer 5 software. If the designed primers could not amplify the first time, we tested other primers and chose the best primers for amplification. The *OTX1* coding sequence was divided into 3 parts (Figure 1(a)) and amplified with PCR using PrimeSTAR® Max DNA Polymerase (Takara, Lot no. R045Q). The PCR reaction tube (50 *μ*l) included 25 *μ*l of the PrimeSTAR Max Premix, 4 *μ*l of primers (0.25 *μ*M), 2 *μ*l of a genomic DNA template (100 ng), and 19 *μ*l of nuclease-free water (Thermo, AM9932) following instructions from the PrimeSTAR® Max DNA Polymerase manual. All amplifications were performed at 98°C. The DNA was denatured for 5 min at 98°C, and then, the cycling continued at 98°C for 10s, 60°C for 15 s, 72°C for 60s (total 32 cycles), and 72°C for 10 min (holding). For the *OTX1* amplification, we also amplified *β*-actin (about 500 bp) as the positive control and used nuclease-free water as the negative control. Fortunately, *β*-actin amplification was universal in three parts of the OTX1 amplification. The PCR products were sent to Shanghai Thermo Fisher Ltd. for Sanger sequencing and analysis. The primer sequences used for PCR and sequencing are listed in Table 1.

### 2.3. Statistical Analysis

Variables were described as the mean and standard deviation or proportion. Differences between participants with and without idiopathic epilepsy were analyzed using chi-square analysis. A *p* value of <0.05 was considered statistically significant. All statistical analyses were performed using SPSS 17 software (IBM, Cary, NC). Power analyses were conducted using Power and Sample Size Calculation Software (http://powerandsamplesize.com/Calculators/). The *OTX1* polymorphism genotype distributions in the control group were tested for their conformity to the Hardy-Weinberg equilibrium (HWE). A *p* value of >0.05 indicated HWE.

## 3. Results

A total of 147 patients with idiopathic epilepsy met inclusion criteria and were included in this study. The mean age was 39.2 years (range 19.8–55.2 years), the female-to-male ratio was 66 : 81, and the mean duration of epilepsy was 4.1 years (range 0–32 years). One hundred and one patients presented with generalized seizures, and 46 patients presented with focal seizures. The demographic and clinical characteristics of the idiopathic epileptic patients are shown in Table 2. One hundred and fifty age- and sex-matched healthy individuals from the same period were recruited to participate in the study as the control group.

After repeated PCR amplification and genotyping, one single-nucleotide polymorphism (SNP) was found in exon-5, and the gene sequence of this site was CACTCACATCACCACCCGCACCAGCT[C/G] AGCCCCATGGCACCCTCCTCCATGC (Figure 1(b)). After blasting this sequence in an SNP database, the SNP number was discovered to be rs17850223.

The distribution of the rs17850223 polymorphism genotypes in the control group was consistent with HWE (*p* > 0.05), indicating that our study population came from a Mendelian population and possessed fine representativeness. As shown in Table 3, further analysis showed that there were no significant differences in the genotype frequency or rs17850223 G allelic distributions between patients with idiopathic epilepsy and control individuals.

When comparing the differences between patients with generalized seizures and the controls, we found that the GG genotype frequency was significantly higher in the patients with generalized seizures compared with those of the controls (12.2% vs. 2%, *p* = 0.012), and there was also a greater distribution of the rs17850223 G allele in the patients with generalized seizures compared with that of the controls (*p* = 0.049) (Table 4). However, no significant differences were found in genotype frequencies or rs17850223 G allelic distributions between the patients with focal seizures and the controls (Table 5).

## 4. Discussion

Epilepsy is a leading neurologic condition triggered by the disruption of abnormal electrochemical activities in the brain. Idiopathic epilepsy, with no apparent structural brain damage or neurologic abnormality, is mainly caused by genetic factors [4–6]. The *OTX1* gene encodes a member of the bicoid subfamily of homeodomain-containing transcription factors. The encoded protein acts as a transcription factor and might play a role in the brain and sensory organ development [15, 16]. Early animal studies indicate that *OTX1* null mice suffered from spontaneous epileptic seizures and exhibited abnormalities that primarily affected the entire dorsal telencephalic cortex with a more pronounced effect in the temporal and perirhinal areas [12, 17]. Recently, Zhang et al. found that *OTX1* expression was essential for the normal development of dendritic morphology, intrinsic electrophysiology, and synaptic dynamics of layer V pyramidal cells in the cerebral cortex [18]. In line with this, another study using mosaic analysis with double markers found that *OTX1* knockouts in cortical progenitors reduce the unitary output of the cortical neurons [19].

Previous basic studies considered that *OTX1* played a crucial role in the development and evolution of the brain, having a protective effect on epilepsy. However, the relationship between the *OTX1* gene and patients with idiopathic epilepsy has not been thoroughly investigated. According to our results, idiopathic epileptic patients with generalized seizures had a significantly greater number of rs17850223 mutations compared with controls. The frequency of the GG genotype and rs17850223 G allele was significantly higher in patients with generalized seizure compared with those of the controls. After blasting the SNP database, the homologous codon was found to be CTC, and some patients with generalized seizures had CTG mutations. However, CTC and CTG encode the same amino acid (L-leucine), which indicated a synonymous mutation.

Owing to the degeneracy of the genetic code, synonymous mutations occurring in the gene coding regions do not change the amino acid composition of the encoded proteins. However, evidence suggests that synonymous mutations could also have functional consequences [20–24] and could result in aberrant mRNA splicing leading to human diseases [20]. Studies also suggested that synonymous SNPs could affect mRNA stability and thus protein expression and enzymatic activity [21]. In addition, it was demonstrated that SNPs could affect protein conformations, which have functional and clinical consequences [22].

Our results indicated that rs17850223 could play a key role in patients with generalized seizures. However, this study had a few limitations. First, the relatively small number of patients could cause false-positive results, especially given the fact that in genetic research, the sample size is a determining factor. Second, we assessed the polymorphisms of *OTX1* coding regions but did not assess the noncoding regions, which could also play a role in idiopathic epilepsy. Furthermore, the identified association should be validated in other populations with different racial and ethnic backgrounds. As this was a preliminary study, additional studies should be performed that look at the whole gene sequence in a larger cohort of patients with different ethnicities to clarify the genetic association between *OTX1* polymorphisms and epilepsy.

In conclusion, this study compared an association between *OTX1* gene polymorphisms and idiopathic epilepsy in Han Chinese Han people. We found that rs17850223 might play a critical role in patients with generalized seizures. The results of this study provided a new understanding regarding the generation of epileptic seizures in patients with idiopathic epilepsy.

## Figures and Tables

**Table 1:** Polymerase chain reaction (PCR) primer sequences of the *OTX1* gene.

*OTX1*	PCR primer sequences	Sequencing primer
*Exon3*-cds	P1 primer: TTCTGTAACCTGCCTTCCC	✔
	P2 primer: CTCACACGCCCACGACTCT	

*Exon4*-cds	P3 primer: GCACTTTCTCCCACCTGT	✔
	P4 primer: AGTCTGTAAGCCCACCCC	

*Exon5*-cds	P5 primer: CTCGGTGAGAAAGGATTG	✔
	P6 primer: GAAGGGGGGAAATAATACAT	

cds: coding sequence.

**Table 2:** Demographic and clinical characteristics of the patients with idiopathic epilepsy.

Variables	Patients (*n* = 147)
Male (*n*, %)	81 (55.1)
Age (years)	39.2 ± 10.7
Duration (years)	4.1 (0–32)
Seizure type	
Partial (*n*, %)	106 (72.1)
Generalized (*n*, %)	41 (27.9)
Family history of epilepsy (*n*, %)	26 (17.7)
Age at first seizure (years)	21.2 (8.2–45.4)
Therapy	
Carbamazepine (*n*, %)	50 (34)
Oxcarbazepine (*n*, %)	38 (25.8)
Valproic acid (*n*, %)	36 (24.5)
Others (*n*, %)	23 (15.6)

Data are shown as mean ± SD, median (range), or *n* (%).

**Table 3:** The distribution of the *OTX1* rs17850223 genotypes in patients with idiopathic epilepsy and the controls.

Variables	Patients (*n* = 147)	Controls (*n* = 150)	*p* value	*P*_HWE_ for controls
Genotype (*n*, %)				0.135
CC	126 (85.7)	124 (82.7)	0.526	
CG	15 (10.2)	23 (15.3)	0.225	
GG	6 (4.1)	3 (2)	0.332	
Allele (*n*, %)			0.781	
C	267 (90.8)	270 (90)		
G	27 (9.2)	30 (10)		

HWE: Hardy-Weinberg equilibrium. Data are shown as *n* (%).

**Table 4:** The distribution of the *OTX1* rs17850223 genotypes in patients with generalized seizures and the controls.

Variables	Generalized seizure (*n* = 41)	Controls (*n* = 150)	*p* value
Genotype (*n*, %)			
CC	31 (75.6)	124 (82.7)	0.367
CG	5 (12.2)	23 (15.3)	0.804
GG	5 (12.2)	3 (2)	0.012^∗^
Allele (*n*, %)			0.049^∗^
C	67 (81.7)	270 (90)	
G	15 (18.3)	30 (10)	

Data are shown as *n* (%); ^∗^*p* < 0.05.

**Table 5:** The distribution of the *OTX1* rs17850223 genotypes in patients with focal seizures and the controls.

Variables	Focal seizure (*n* = 106)	Controls (*n* = 150)	*p* value
Genotype (*n*, %)			
CC	95 (89.6)	124 (82.7)	0.149
CG	10 (9.4)	23 (15.3)	0.189
GG	1 (0.9)	3 (2)	0.644
Allele (*n*, %)			1.000
C	190 (90)	270 (90)	
G	21 (10)	30 (10)	

Data are shown as *n* (%).

## Data Availability

The data used to support the findings of this study are included within the article.
